# Evaluation of the pharmacoDYNAMIC effects of riociguat in subjects with pulmonary hypertension and heart failure with preserved ejection fraction: Study protocol for a randomized controlled trial

**DOI:** 10.1007/s00508-016-1068-8

**Published:** 2016-09-02

**Authors:** Julia Mascherbauer, Ekkehard Grünig, Michael Halank, Wolfgang Hohenforst-Schmidt, Andreas A. Kammerlander, Ingrid Pretsch, Regina Steringer-Mascherbauer, Silvia Ulrich, Irene M. Lang, Manfred Wargenau, Reiner Frey, Diana Bonderman

**Affiliations:** 1 Department of Cardiology, Medical University of Vienna, Waehringer Guertel 18–20, 0190 Vienna, Austria; 2 Centre for Pulmonary Hypertension, Thoraxclinic, University Hospital of Heidelberg, Heidelberg, Germany; 3 Department of Internal Medicine I, University Hospital Carl Gustav Carus Dresden, Dresden, Germany; 4 Medical Clinic II, Coburg Hospital, Coburg, Germany; 5 Department of Cardiology, Paracelsus Medial University Salzburg, Salzburg, Austria; 6 Department of Cardiology, Public Hospital Elisabethinen Linz, Linz, Austria; 7 Pulmonary Clinic, Department of Cardiovascular and Thoracic Medicine, University Hospital Zurich, Zurich, Switzerland; 8 M.A.R.C.O. GmbH & Co. KG, Düsseldorf, Germany; 9 Am Eckbusch 75, 42113 Wuppertal, Germany

**Keywords:** Heart failure with preserved ejection fraction, Pulmonary hypertension, Medical treatment

## Abstract

**Background:**

The presence of pulmonary hypertension (PH) severely aggravates the clinical course of heart failure with preserved ejection fraction (HFPEF) resulting in substantial morbidity and mortality. So far, neither established heart failure therapies nor pulmonary vasodilators have proven to be effective for this condition. Riociguat (Adempas®, BAY 63-2521), a stimulator of soluble guanylate cyclase, is a novel pulmonary and systemic vasodilator that has been approved for the treatment of precapillary forms of PH. With regard to postcapillary PH, the DILATE-1 study was a multicenter, double-blind, randomized, placebo-controlled single-dose study in subjects with PH associated with HFPEF. Although there was no significant change in the primary outcome measure, peak decrease in mean pulmonary artery pressure with riociguat versus placebo, riociguat significantly increased stroke volume without changing heart rate, pulmonary artery wedge pressure, transpulmonary pressure gradient or pulmonary vascular resistance. The present study is designed to test the efficacy of long-term treatment with riociguat in patients with PH associated with HFPEF.

**Methods/study design:**

The DYNAMIC study is a randomized, double-blind, placebo-controlled, parallel-group, multicenter clinical phase IIb trial evaluating the efficacy, safety and kinetics of riociguat in PH-HFPEF patients. The drug will be given over 26 weeks to evaluate the effects of riociguat versus placebo. The primary efficacy variable will be the change from baseline in cardiac output at rest, measured by right heart catheter after 26 weeks of study drug treatment. Additional efficacy variables will be changes from baseline in further hemodynamic parameters, changes in left and right atrial area, right ventricular volume, as well as right ventricular ejection fraction measured by cardiac magnetic resonance imaging, and changes from baseline in World Health Organization (WHO) class and N‑terminal prohormone B‑type natriuretic peptide (NT-proBNP).

The trial was registered on 25 August 2014 (EudraCT Number: 2014-003055-60; www.clinicaltrialsregister.eu).

## Background

Nearly half of the patients presenting with symptoms of heart failure have a normal left ventricular ejection fraction [1]. This condition is called heart failure with preserved ejection fraction (HFPEF) and also known as diastolic heart failure. The major determinant of mortality in this patient population is coexisting pulmonary hypertension (PH) [2], classified as group 2.2 in the Dana Point classification [3]. Despite the frequency and severity of HFPEF, effective medical treatment is lacking. sRiociguat (Adempas®, BAY 63-2521), an oral stimulator of soluble guanylate cyclase, is a novel pulmonary and systemic vasodilator that has been approved for precapillary forms of PH, e.g. chronic thromboembolic pulmonary hypertension (CTEPH) [4] and pulmonary arterial hypertension (PAH) [5]. Two phase II trials have indicated a potential benefit of riociguat treatment also in patients with postcapillary PH due to left heart disease. The phase IIb Left ventricular systolic dysfunction associated with pulmonary hypertension riociguat trial (LEPHT) study was a multicenter, double-blind, randomized, placebo-controlled study in subjects with PH resulting from heart failure with reduced ejection fraction (HFREF) [4, 6]. A total of 201 subjects were randomized to treatment with oral placebo or riociguat (0.5, 1 or 2 mg TID) for 16 weeks in 4 parallel arms. The primary outcome was the placebo-corrected change from baseline at week 16 in mean pulmonary artery pressure (PAPmean). Although the decrease in PAPmean in the riociguat 2 mg group was not significantly different from placebo, cardiac index and stroke volume index were significantly increased without changes in heart rate (HR) or systolic blood pressure (SBP) compared with placebo. Both pulmonary and systemic vascular resistance (PVR and SVR) were significantly reduced with 2 mg riociguat TID. Riociguat reduced the Minnesota Living with Heart Failure (MLHF) score.

The proof of concept study DILATE-1 was a multicenter, double-blind, randomized, placebo-controlled single-dose study in subjects with PH associated with HFPEF [7]. Clinically stable subjects with a left ventricular ejection fraction (LVEF) >50 %, PAPmean ≥25 mm Hg and pulmonary arterial wedge pressure (PAWP) >15 mm Hg at rest were randomized to single oral doses of placebo or riociguat. There were no significant changes in peak decrease in PAPmean with riociguat 2 mg versus placebo; however, riociguat 2 mg significantly increased stroke volume and decreased SBP and right ventricular end-diastolic area, without significantly changing HR, PAWP, the transpulmonary pressure gradient (TPG) and PVR. Based on the results of the single-dose DILATE-1 study in PH-HFPEF patients and the LEPHT study over 16 weeks in subjects with PH-HFREF, the present study is expected to generate data to assess the therapeutic potential of riociguat in PH-HFPEF.

## Study design

The DYNAMIC study is a randomized, double-blind, placebo-controlled, parallel-group, multicenter clinical phase IIb trial evaluating the hemodynamic effects, safety, and kinetics of riociguat in PH-HFPEF patients. The study drug will be administered over 26 weeks to evaluate the effects of riociguat versus placebo. Written informed consent will be obtained from patients in accordance with the Declaration of Helsinki and the ethics committee of the Medical University of Vienna has approved the study protocol (date of vote: 10-OCT-2014; reference number: EK 1570/2014).

## Titration and treatment phases

**Fig. 1:**
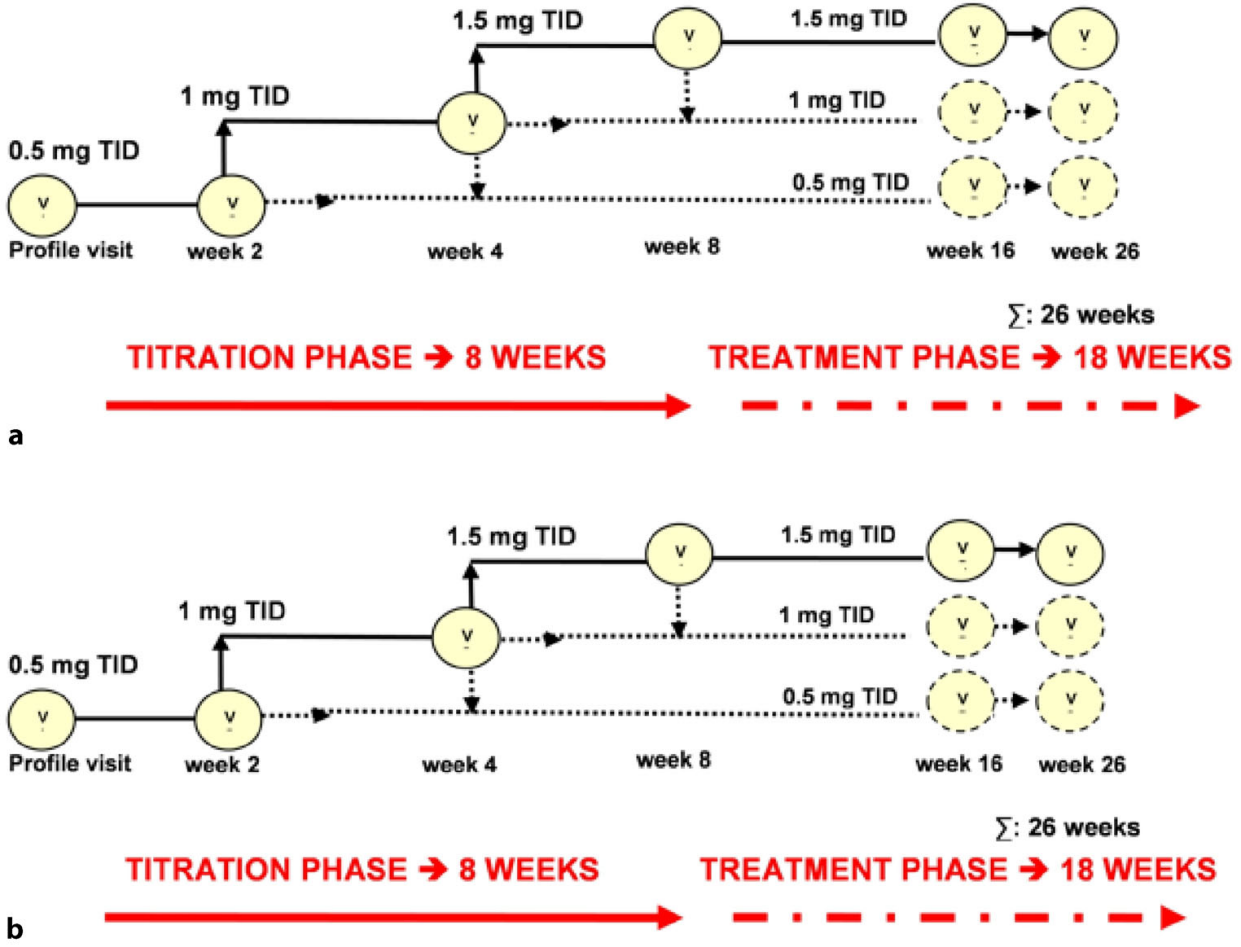
Scheme of up-titration regimen in the DYNAMIC study. *TID* 3 times a day, *v* study visit. **a** up-titration of riociguat up to 1.5 mg TID. **b** sham titration placebo TID

After a pretreatment phase of up to 4 weeks, the study phase will consist of an 8‑week (up-)titration phase followed by the 18-week fixed-dose treatment. Patients will be randomized to the active treatment arm or placebo. The starting dose of riociguat will be 0.5 mg 3 times a day (TID). At visits 2 and 3 (weeks 2 and 4), up-titration to a dose of 1.0 and 1.5 mg TID (Fig. 1) is based on clinical condition and systemic SBP, measured before intake of the next study drug dose in accordance with the following algorithm:

During the titration phase in the first 8 weeks, the dose for the next titration step will depend on the SBP measured at trough before intake of the next study drug dose. The following algorithm applies to the individual dose titration (IDT) scheme:

- If trough SBP is ≥110 mm Hg, increase the dose.
- If trough SBP is between 100 and 109 mm Hg without symptoms of hypotension, maintain the dose.
- If trough SBP is <100 mm Hg without symptoms of hypotension, reduce the dose.

The IDT scheme is based on blood pressure and consideration of the subject’s clinical condition; therefore, in cases of poor tolerability, a planned up-titration step may be postponed and the dose maintained. During the 18-week treatment phase (from visit 3 to visit 6), all patients will receive the dose of visit 3. Dose reductions or interruptions for safety reasons are allowed at any time.

## Selected patient enrolment criteria

Eligible patients will be aged 18–80 years at the time of informed consent and have symptomatic PH-HFPEF, e.g. group 2/2.2 of the Dana Point classification [3] and World Health Organization (WHO) class II to IV, defined as:

- LVEF ≥50 %, diagnosed by echocardiography or left heart catheterization (LHC) within 30 days before randomization,
- PAPmean ≥25 mm Hg at rest, measured by right heart catheter (RHC) within 12 weeks before randomization,
- PAWP >15 mm Hg at rest, measured by RHC within 12 weeks before randomization.

The dose regimen of the background treatment must have been stable for >30 days before randomization. Diuretic therapy must have been stable for ≥1 week.

Selected exclusion criteria are listed in Table 1.

**Table 1:** Selected exclusion criteria

Cardiac decompensation, either with hospitalization or visit to the emergency department ≤30 days before randomization
Resynchronization therapy
Need of IV diuretics ≤30 days before randomization
Treatment with IV inotropes or IV vasodilators ≤30 days before randomization
Chronic treatment with ERAs, PDE-5 inhibitors or prostanoids ≤30 days before randomization or with nitrates or PDE-5 inhibitors indicated for erectile dysfunction ≤7 days before randomization
Bronchial asthma or COPD with FEV_1_ <60 % of predicted
Restrictive lung disease with TLC <60 % of predicted
Current O_2_ therapy
Clinically relevant hepatic dysfunction indicated by either AST ≥3 times the upper limit of normal or Child-Pugh stage B and C in patients with cirrhosis
Severe renal impairment (glomerular filtration rate <30 ml/min/1.73 m^2^ calculated by the Modification of Diet in Renal Disease formula)
Uncontrolled arterial hypertension (SBP >180 mm Hg or DBP >110 mm Hg)
SBP <100 mm Hg at baseline or clinical signs or symptoms of hypotension
Myocardial disease, such as infiltrative myocardial disease (i. e. amyloidosis, hypertrophic cardiomyopathy)
Severe aortic or mitral stenosis or regurgitation or any valvular stenosis or regurgitation with indications for surgery
Coronary artery disease with angina of Canadian Cardiovascular Society class III or IV or requiring nitrates
Unstable angina or acute myocardial infarction <90 days before randomization
Reperfusion procedure (PCI or coronary artery bypass graft) <90 days prior to randomization or <3 weeks in case of a negative stress test after PCI
Stroke with persistent neurological deficits or known hemodynamically relevant symptomatic carotid artery stenosis
Resting heart rate while awake of <50 BPM or >105 BPM or in the case of AF >110 BPM

*IV* indicates intravenous, *ERAs* endothelin receptor antagonists, *PDE-5* phosphodiesterase-5, *COPD* chronic obstructive pulmonary disease, *FEV*_1_ forced expiratory volume in 1 s, *TLC* total lung capacity, *O*_2_ oxygen, *AST* aspartate transaminase, *SBP* systolic blood pressure, *DBP* diastolic blood pressure, *PCI* percutaneous coronary intervention, *BPM* beats per minute, *AF* atrial fibrillation

## Study objectives

The primary objective of this study is to assess the pharmacodynamic profile of riociguat in subjects with symptomatic PH-HFPEF. The secondary objectives of this study are to assess changes in dimensions of left and right ventricles and cardiac function parameters using cardiac magnetic resonance imaging (CMRI). Patients with a PVR above and below 240 dyn × s × cm^−5^ will be analyzed as prespecified subgroups.

## Primary efficacy variable

The primary efficacy variable is the change from baseline of cardiac output (CO) at rest, measured by RHC after 26 weeks of study drug treatment.

## Secondary efficacy variables

- Change from baseline in left and right atrial area, right ventricular volume and right ventricular ejection fraction, measured by CMRI
- Change from baseline in PVR, SVR, TPG and PAWP, measured by RHC
- Change from baseline in WHO class
- Change from baseline in serum N‑terminal prohormone B‑type natriuretic peptide (NTproBNP) levels

## Exploratory efficacy variables

- Change from baseline in all other CMRI parameters not listed above. At selected sites only: T1 mapping and extracellular volume (ECV).
- Change from baseline in all other RHC parameters not listed under secondary variables.
- Change from baseline in echocardiography parameters, including left ventricular end-diastolic and end-systolic volumes, tricuspid annular plan systolic excursion, pressure gradient of tricuspid valve, diameter of inferior vena cava, respiratory collapsibility of inferior vena cava, mitral peak velocity of early (E) and late (A) filling, E‑wave deceleration time, LVEF, estimate of mean right atrial pressure, systolic pulmonary artery pressure and E/A ratio.
- Change from baseline in exercise capacity (6 min walk distance) and in Borg CR 10 scale.
- Change from baseline in quality of life scores EuroQol five dimensions questionnaire (EQ-5D), MLHF.
- Events of special interest considered for calculation of the combined endpoint time to clinical worsening.
- All-cause mortality.
- Composite endpoint as defined by time to death from cardiovascular causes or first hospitalization for a cardiovascular event, including acute or worsening heart failure, acute myocardial infarction, stroke or ventricular arrhythmia.
- Change in glomerular filtration rate.

## Pharmacokinetics

To investigate drug exposure and potential relationships to drug effects, plasma concentrations of riociguat and its main metabolite M‑1 (BAY 60-4552) will be determined. Plasma concentrations will be measured using a validated high performance liquid chromatography-mass spectrometry/tandem mass spectrometry (HPLC-MS/MS) method. Quality control (QC) and calibration samples will be analyzed concurrently with study samples. The results of QC samples will be reported together with concentrations in unknown samples in the clinical study report.

## Statistical considerations

Statistical analyses will be performed using Statistical Analysis Software (SAS®). A randomized subject will be valid for safety analysis if at least one dose of study drug was administered. A subject will be part of the full analysis set (FAS) in accordance to the International Conference on Harmonization (ICH) E9 guideline, if he/she belongs to the safety set, is not violating any major entry criteria and shows any postbaseline efficacy data. A subject will be valid for the per-protocol analysis if the subject belongs to FAS and does not show any major protocol deviations that interfere with the interpretation of efficacy results. All variables will be analyzed descriptively with appropriate statistical methods: categorical variables by frequency tables and continuous variables by sample statistics (e.g. number of non-missing data, mean, standard deviation, minimum, median, quartiles and maximum).

The primary efficacy variable will be submitted to an analysis of covariance (ANCOVA) model including the baseline value as covariate, center, treatment regimen and the interaction term of center by treatment regimen as fixed effects. If the treatment-center interaction is not significant at the 0.2 level, the interaction term will be removed from the model for final analysis. The primary comparison will be a one-sided test at the 2.5 % significance level for the difference in treatment effects between riociguat and placebo.

Continuous secondary endpoints will be analyzed in the same manner as the primary endpoint. The change of WHO class will be dichotomized by defining the groups: “improvement by at least one class”, “no improvement by at least one class” and analyzed using Mantel-Haenszel weights stratified by center. The secondary efficacy variables will be tested in exploratory manner at nominal one-sided significance level of 2.5 %.

### Determination of sample size

Requiring an 80 % power of the trial to detect a mean difference of at least 0.6 l/min between riociguat and placebo with regard to the primary endpoint and assuming a standard deviation of 1.0 l/min, which is derived from a previous trial (LEPHT), then 45 evaluable subjects are needed in each of the 2 treatment groups. Taking into account a drop-out rate of 20 %, a total of 114 subjects are to be randomized (57 subjects per group).

## Discussion

The DYNAMIC study is the first randomized, double-blind, placebo-controlled, multi-center trial testing the effects of an oral soluble guanylate cyclase (sGC) stimulator targeting PH due to HFPEF. The results will potentially be of great clinical relevance given the high prevalence of PH among patients with HFPEF and its influence on morbidity and mortality.

### Pulmonary vasodilators in heart failure with preserved ejection fraction

Pulmonary vasodilators have previously been studied in only few clinical trials, which have yielded conflicting results. In a placebo-controlled, single center clinical study in PH-HFPEF patients, the phosphodiesterase-5 inhibitor sildenafil lowered pulmonary artery pressure and improved right ventricular function [8]. By contrast, sildenafil failed to improve endpoints in a cohort of HFPEF patients without significant PH [9]. In the RELAX trial [9] peak O_2_ consumption was not changed in the treatment arm as compared with placebo. Secondary endpoints including change in 6 min walking distance, composite clinical status score and change in quality of life at 24 weeks were also missed. Moreover, subgroup analysis showed no improvement in peak O_2_ consumption in patients with higher pulmonary artery systolic pressure where the median was 41 mm Hg (range 32–51 mm Hg) in the treatment group.

Currently, two additional multicenter, placebo-controlled clinical trials testing the effects of pulmonary vasodilators in HFPEF are underway. The SOCRATES PRESERVED program was designed to study the effects of the novel once-daily oral sGC stimulator vericiguat (BAY 1021189) in a phase II dose-finding study among patients with HFPEF stabilized after hospitalization or i. v. diuretic therapy for worsening of chronic heart failure [10]. Although the DYNAMIC study follows a similar study rationale that is based on systemic vasoreactivity and potential cardiac effects of the sGC stimulator, main differences with respect to SOCRATES PRESERVED are the patient population under investigation (HFPEF versus PH-HFPEF) and main study endpoints (change in NT-proBNP and left atrial volume at 12 weeks versus invasively measured CO at 26 weeks).

The MELODY study, which has recently been rolled out, focuses on the effects of macitentan, a novel dual endothelin receptor blocker versus placebo in a rare subpopulation of PH-HFPEF patients, which are characterized by elevated pulmonary diastolic pressure gradients indicative of true pulmonary vascular remodelling on top of elevated left-sided filling pressures; however, there is cumulating evidence that the use of riociguat in patients with PH associated with heart failure is most effective in the subgroup with a PVR <240 dyn ×s ×cm^−5^ [11]. Given the uncertainties regarding the optimal target population, i. e. pure postcapillary versus combined precapillary and postcapillary PH, the current protocol will be open for a broad range of hemodynamic profiles of patients fulfilling the diagnostic criteria of postcapillary PH.

### Rationale for the long-term use of riociguat in pulmonary hypertension due to heart failure with preserved ejection fraction

Riociguat is a direct oral sGC stimulator [12] that targets reduced nitric oxide (NO) bioavailability in PH as well as in heart failure. Riociguat stimulates the enzymatic activity of sGC to generate cyclic guanosine monophosphate (cGMP) both independently of NO and synergistically with NO, and is a potent, dose-dependent vasodilator [13]. Beyond its hemodynamic effects, riociguat reduced renal and cardiac fibrosis, increased creatinine clearance, decreased atrial natriuretic peptide and left ventricular mass in two independent models of hypertension [14].

More recently, the beneficial effect of riociguat on hemodynamic and clinical endpoints has been demonstrated in three placebo-controlled trials. Riociguat significantly improved exercise capacity and secondary efficacy endpoints in the phase III Pulmonary Arterial Hypertension Soluble Guanylate Cyclase-Stimulator Trial (PATENT) [5] and the Chronic Thromboembolic Pulmonary Hypertension Soluble Guanylate Cyclase-Stimulator Trial (CHEST) [4]. In a randomized, placebo-controlled phase IIb study in patients with heart failure and PH due to systolic left ventricular dysfunction (LEPHT) [4], riociguat was well-tolerated and improved the cardiac index, PVR and SVR, as well as quality of life, without significantly changing PAPmean (primary endpoint) or SBP.

Taken together, the efficacy of long-term treatment with riociguat has been demonstrated in precapillary as well as postcapillary PH. Given similar pathophysiological changes, such as elevation in left ventricular filling pressures (LEPHT) [4] and PVR (PATENT and CHEST) [4, 5] in PH-HFPEF, we speculate that previously observed beneficial effects of riociguat on pulmonary and systemic vascular trees as well as cardiac function will also occur in PH-HFPEF. This is supported by recently published results from the DILATE-1 (Acute Hemodynamic Effects of Riociguat in Patients with Pulmonary Hypertension Associated with Diastolic Heart Failure [7]) study, a multicenter, placebo-controlled phase IIa trial that tested safety and efficacy of single oral doses of riociguat (0.5, 1.0 or 2.0 mg) in PH-HFPEF. There was no significant change in peak decrease in PAPmean (primary endpoint) with 2 mg riociguat versus placebo; however, 2 mg riociguat significantly increased stroke volume and decreased SBP and right ventricular end-diastolic area, without significantly changing heart rate, PAWP, TPG or PVR. Importantly, riociguat was well tolerated.

### Change in cardiac output as primary endpoint

The primary objective of the present study is to test the effects of riociguat on the surrogate endpoint CO. While CO is one of the established therapy response markers in PH [3], therapeutic interventions that lead to an increase in CO in left heart disease are under debate. While an increase in CO based on a positive inotropic effect results in elevated left ventricular filling pressures and increased mortality [15], in the LEPHT study [4], which was conducted in heart failure patients, riociguat significantly improved CO in the absence of any significant change in heart rate or SBP compared with placebo. Most importantly, left ventricular filling pressures remained unchanged without excess mortality and clinical benefit was supported by an improvement in quality of life. The mechanisms underlying the observed increase in CO in the LEPHT study are complex. Improvements were not attributable to reflex sympathetic activation elicited by a decrease in SBP. The rise in cardiac function was paralleled by a decrease in PVR and SVR. The potential of direct sGC stimulators in heart failure might go beyond hemodynamic effects and additionally, or even predominantly, rely on non-hemodynamic effects of sGC-derived cGMP signalling in a variety of tissues including cardiac myocytes [16]. Furthermore, preclinical data suggest that sGC stimulation does not increase cyclic adenosine monophosphate (cAMP) production despite significant cGMP increases and may blunt the inotropic response to adrenergic stimulation [17, 18]. In the DILATE-1 study a pronounced effect of riociguat on CO was observed accompanied by size reduction of the right ventricle. These data provide a strong rationale for CO as a primary endpoint in PH-HFPEF patients.

#### Cardiac magnetic resonance imaging substudy

Patients in all centers will undergo a CMRI study. Studies will consist of functional and late gadolinium enhancement (LGE) imaging, according to standard protocols [19]. In the Vienna subgroup T1 mapping sequences [20] will be used for the quantification of ECV in the left ventricular myocardium. This technique has been developed for characterization and quantification of subtle diffuse myocardial fibrosis [20]. In light of potential direct, non-hemodynamic effects of riociguat on the myocardium, this substudy may provide insights into alternative mechanisms of action.

## Conclusion

- Based on experiences from the DILATE-1, LEPHT, PATENT and CHEST trials, the DYNAMIC study adds innovative principles to a phase II drug development program in PH-HFPEF.
- Given the previously reported pharmacodynamic activity of the novel drug class of oral sGC stimulators in patients with heart failure and patients with PH, the study design provides the groundwork for a subsequent phase III pivotal trial in patients with PH-HFPEF.

## Abbreviations

CTEPH: Chronic thromboembolic pulmonary hypertension
DPG: Diastolic pressure gradient
HFPEF: Heart failure with preserved ejection fraction
HR: Heart rate
IDT: Individual dose titration
LVEF: Left ventricular ejection fraction
MLHF: Minnesota living with heart failure score
PAH: Pulmonary arterial hypertension
PAPmean: Mean pulmonary artery pressure
PAWP: Pulmonary arterial wedge pressure
PH: Pulmonary hypertension
PVR: Pulmonary vascular resistance
RHC: Right heart catheter
SBP: Systolic blood pressure
sGC: Soluble guanylate cyclase
SVR: Systemic vascular resistance
TID: Ter in die (three times daily)
TPG: Transpulmonary pressure gradient

## References

[1] McMurray JJ, Adamopoulos S, Anker SD (2012). ESC guidelines for the diagnosis and treatment of acute and chronic heart failure 2012: the task force for the diagnosis and treatment of acute and chronic heart failure 2012 of the European Society of Cardiology. Developed in collaboration with the Heart Failure Association (HFA) of the ESC. Eur. Heart J..

[2] Lam CS, Roger VL, Rodeheffer RJ, Borlaug BA, Enders FT, Redfield MM (2009). Pulmonary hypertension in heart failure with preserved ejection fraction: a community-based study. J. Am. Coll. Cardiol..

[3] Galie N, Hoeper MM, Humbert M (2009). Guidelines for the diagnosis and treatment of pulmonary hypertension: the task force for the diagnosis and treatment of pulmonary hypertension of the European Society of Cardiology (ESC) and the European Respiratory Society (ERS), endorsed by the International Society of Heart and Lung Transplantation (ISHLT). Eur. Heart J..

[4] Bonderman D, Ghio S, Felix SB (2013). Riociguat for patients with pulmonary hypertension caused by systolic left ventricular dysfunction: a phase IIb double-blind, randomized, placebo-controlled, dose-ranging hemodynamic study. Circulation.

[5] Ghofrani HA, Galie N, Grimminger F (2013). Riociguat for the treatment of pulmonary arterial hypertension. N. Engl. J. Med..

[6] Ghio S, Bonderman D, Felix SB (2012). Left ventricular systolic dysfunction associated with Pulmonary Hypertension riociguat Trial (LEPHT): rationale and design. Eur. J. Heart Fail..

[7] Bonderman D, Pretsch I, Steringer-Mascherbauer R (2014). Acute hemoDynamic effects of rIociguat in patients with puLmonary hypertension Associated with diasTolic heart failurE (DILATE-1)): a randomized, double-blind, placebo-controlled, single-dose study. Chest.

[8] Guazzi M, Vicenzi M, Arena R, Guazzi MD (2011). Pulmonary hypertension in heart failure with preserved ejection fraction: a target of phosphodiesterase-5 inhibition in a 1‑year study. Circulation.

[9] Redfield MM, Chen HH, Borlaug BA (2013). Effect of phosphodiesterase-5 inhibition on exercise capacity and clinical status in heart failure with preserved ejection fraction: a randomized clinical trial. JAMA.

[10] Pieske B, Butler J, Filippatos G (2014). Rationale and design of the SOluble guanylate Cyclase stimulatoR in heArT failurE Studies (SOCRATES). Eur. J. Heart Fail..

[11] Bonderman D, Ghio S, Felix SB, et al. Riociguat for heart failure with secondary pulmonary hypertension: post-hoc analysis of the LEPHT study. Eur Heart J Suppl. 2014;35:385

[12] Stasch JP, Evgenov OV (2013). Soluble guanylate cyclase stimulators in pulmonary hypertension. Handb Exp Pharmacol.

[13] Stasch JP, Becker EM, Alonso-Alija C (2001). NO-independent regulatory site on soluble guanylate cyclase. Nature.

[14] Sharkovska Y, Kalk P, Lawrenz B (2010). Nitric oxide-independent stimulation of soluble guanylate cyclase reduces organ damage in experimental low-renin and high-renin models. J. Hypertens..

[15] Packer M, Carver JR, Rodeheffer RJ (1991). Effect of oral milrinone on mortality in severe chronic heart failure. The PROMISE Study Research Group. N. Engl. J. Med..

[16] Paulus WJ, Tschope C (2013). A novel paradigm for heart failure with preserved ejection fraction: comorbidities drive myocardial dysfunction and remodeling through coronary microvascular endothelial inflammation. J. Am. Coll. Cardiol..

[17] Cawley SM, Kolodziej S, Ichinose F, Brouckaert P, Buys ES, Bloch KD (2011). sGC{alpha}1 mediates the negative inotropic effects of NO in cardiac myocytes independent of changes in calcium handling. Am J Physiol Heart Circ Physiol.

[18] Ramos-Espiritu LS, Hess KC, Buck J, Levin LR (2011). The soluble guanylyl cyclase activator YC-1 increases intracellular cGMP and cAMP via independent mechanisms in INS-1E cells. J. Pharmacol. Exp. Ther..

[19] Kramer CM, Barkhausen J, Flamm SD, Kim RJ, Nagel E (2013). Standardized cardiovascular magnetic resonance (CMR) protocols 2013 update. J Cardiovasc Magn Reson.

[20] Moon JC, Messroghli DR, Kellman P (2013). Myocardial T1 mapping and extracellular volume quantification: a Society for Cardiovascular Magnetic Resonance (SCMR) and CMR Working Group of the European Society of Cardiology consensus statement. J Cardiovasc Magn Reson.

